# Analysis of distribution, collection, and confirmation of capacity dependency of small extracellular vesicles toward a therapy for liver cirrhosis

**DOI:** 10.1186/s41232-023-00299-x

**Published:** 2023-10-09

**Authors:** Nobutaka Takeda, Atsunori Tsuchiya, Masaki Mito, Kazuki Natsui, Yui Natusi, Yohei Koseki, Kei Tomiyoshi, Fusako Yamazaki, Yuki Yoshida, Hiroyuki Abe, Masayuki Sano, Taketomo Kido, Yusuke Yoshioka, Junichi Kikuta, Tohru Itoh, Ken Nishimura, Masaru Ishii, Takahiro Ochiya, Atsushi Miyajima, Shuji Terai

**Affiliations:** 1 Division of Gastroenterology and Hepatology, Graduate School of Medical and Dental Sciences, Niigata University, 1-757 Asahimachi-Dori, Chuo-Ku, Niigata, 951-8510 Japan; 2 Future Medical Research Center for Exosome and Designer Cell (F-EDC), Niigata University, 1-757 Asahimachi-Dori, Chuo-Ku, Niigata, 951-8510 Japan; 3 Cellular and Molecular Biotechnology Research Institute, National Institute of Advanced Industrial Science and Technology (AIST), Central 5, 1-1-1 Higashi, Tsukuba, Ibaraki 305-8565 Japan; 4 Laboratory of Stem Cell Therapy, Institute for Quantitative Biosciences, University of Tokyo, Tokyo, 113-0032 Japan; 5 Department of Molecular and Cellular Medicine, Institute of Medical Science, Tokyo Medical University, 6-7-1, Nishi-Shinjuku, Shinjuku-Ku, Tokyo, 160-0023 Japan; 6 Department of Immunology and Cell Biology, Graduate School of Medicine and Frontier Biosciences, Osaka University, 2-2 Yamada-Oka, Suita, Osaka 565-0871 Japan; 7 Laboratory of Gene Regulation, Institute of Medicine, University of Tsukuba, 1-1-1 Tennodai, Tsukuba, Ibaraki 305-8575 Japan

**Keywords:** Extracellular vesicle, Liver cirrhosis, Tangential flow filtration, Macrophage

## Abstract

**Background:**

The progression of liver fibrosis leads to portal hypertension and liver dysfunction. However, no antifibrotic agents have been approved for cirrhosis to date, making them an unmet medical need. Small extracellular vesicles (sEVs) of mesenchymal stem cells (MSCs) are among these candidate agents. In this study, we investigated the effects of sEVs of MSCs, analyzed their distribution in the liver post-administration, whether their effect was dose-dependent, and whether it was possible to collect a large number of sEVs.

**Methods:**

sEVs expressing tdTomato were generated, and their uptake into constituent liver cells was observed in vitro, as well as their sites of uptake and cells in the liver using a mouse model of liver cirrhosis. The efficiency of sEV collection using tangential flow filtration (TFF) and changes in the therapeutic effects of sEVs in a volume-dependent manner were examined.

**Results:**

The sEVs of MSCs accumulated mostly in macrophages in damaged areas of the liver. In addition, the therapeutic effect of sEVs was not necessarily dose-dependent, and it reached a plateau when the dosage exceeded a certain level. Furthermore, although ultracentrifugation was commonly used to collect sEVs for research purposes, we verified that TFF could be used for efficient sEV collection and that their effectiveness is not reduced.

**Conclusion:**

In this study, we identified some unknown aspects regarding the dynamics, collection, and capacity dependence of sEVs. Our results provide important fundamentals for the development of therapies using sEVs and hold potential implications for the therapeutic applications of sEV-based therapies for liver cirrhosis.

**Supplementary Information:**

The online version contains supplementary material available at 10.1186/s41232-023-00299-x.

## Background

Cirrhosis is a result of liver damage with inflammation and subsequent fibrosis caused by viruses, such as hepatitis B and C, alcohol, and Western lifestyle habits. The pathogenesis includes portal hypertension and decreased liver function, which are associated with fibrosis [1]. As portal hypertension and hepatic dysfunction progress, various symptoms such as bleeding, jaundice, edema, ascites, hepatic encephalopathy, and increased infections occur owing to changes in circulatory dynamics, invasion of intestinal bacteria and components, systemic inflammation, immune system changes, and metabolic and nutritional disorders, which significantly reduce the quality of life and lead to fatal complications in patients. Liver transplantation is the ultimate treatment; however, donor shortage poses a serious challenge [2, 3].

Previous studies have shown that mesenchymal stem cells (MSCs) can improve fibrosis and indirectly promote regeneration by acting on tissue repair macrophages, which are involved in fibrosis and hepatocyte regeneration at the site of liver injury [4]. Furthermore, MSCs can induce this effect through small extracellular vesicles (sEVs) approximately 100 nm in size. sEVs contain a large number of proteins and nucleic acids and have recently attracted attention for their use as not only biomarkers but also therapeutic agents [5]. In particular, we have shown that sEVs collected after IFN-γ stimulation of MSCs have a strong effect on macrophages in the damaged area of the liver, inducing anti-inflammatory macrophages with high motility and phagocytic activity and that they exert a strong therapeutic effect against liver cirrhosis [6]. Thus, sEVs emerge as a promising novel candidate for the treatment of liver cirrhosis.

However, several challenges remain in the development of therapies using MSC-derived sEVs in targeting the unmet medical needs for liver cirrhosis, namely improving fibrosis and promoting regeneration. For example, it remains unclear which cells in the liver sEVs are distributed to after administration, whether they act in a dose-dependent manner, and whether it is possible to collect a large number of sEVs. In this study, we conducted basic research to address these challenges in the development of sEV-based therapies for liver cirrhosis.

## Methods

### Mice

C57BL/6 male and female mice were purchased from Charles River (Yokohama, Japan). The mice were housed in a specific pathogen-free environment, maintained under standard conditions with a 12-h day/night cycle, and had ad libitum access to food and water. All animal experiments were conducted in compliance with the regulations of Niigata University and were approved by the Institutional Animal Care Committees.

### Human MSCs

Human adipose tissue-derived MSCs (AD-MSCs; passage 2) were obtained from PromoCell (Heidelberg, Germany; catalog number C-12977) and expanded until passage 4 using Cellartis MSC Xeno-Free Culture Medium (Takara Bio, Kusatsu, Japan) in low oxygen (5% O_2_) and in the presence of 5% CO_2_ at 37 °C. Cells were tested by PromoCell for morphology, proliferation potential, adherence rate, and viability. Cells were analyzed by flow cytometry using a comprehensive panel of markers: CD73/CD90/CD105 and CD14/CD19/CD34/CD45/HLA-DR. Adipogenic, osteogenic, and chondrogenic differentiation assays were performed for each lot in the absence of antibiotics and antimycotics.

### Production and infection of Sendai virus vectors

cDNAs encoding tdTomato and human CD63 were amplified by PCR from Ai9 (#22799; Addgene) and CD63-pEGFP C2 (#62964; Addgene), respectively, and were fused using an In-Fusion HD Cloning Kit (TaKaRa Bio) to develop the hCD63-tdTomato construct. The hKO gene was deleted from the SeVdp(*Bsr/∆F/hKO*) vector cDNA [7], and hCD63-tdTomato was inserted before the Bsr gene to produce the SeVdp (CD63TB) vector cDNA. Sendai virus vector (CD63TB) was prepared from the vector cDNA as described previously and infected to human MSCs at 32˚C for 14–16 h. To select vector-infected cells, 20 µg/mL blasticidin S was added 2 days after infection. The oligonucleotide sequences used for vector construction are listed in Table 1.

**Table 1:** Oligonucleotide sequences for plasmid construction

**cDNA amplification**	
hCD63	5′accgctagcacctaggtctgacaccATGGCGGTGGAAGGAGGAATG-3′
	5′-CATCACCTCGTAGCCACTTCTGATAC-3′
tdTomato	5′-ggctacgaggtgatgGTGAGCAAGGGCGAGGAGG-3′
	5′-agtttttcttaatcgacgtctTTACTTGTACAGCTCTGTCCATGCC-3′

### Collection of sEVs (ultracentrifuge)

Passage 4 AD-MSC were cultured without serum in advanced Dulbecco’s modified Eagle’s medium (DMEM) (Thermo Fisher Scientific) with GlutaMAX™ (100 ×) (Thermo Fisher Scientific) for 48 h. The supernatant was collected and centrifuged at 2000 × *g* for 10 min to remove cell debris and filtered using a 0.22-μm filter (Stericup™ Quick Release Durapore™, Merck Millipore, Burlington, MA, USA). The filtered supernatant was ultracentrifuged at 210,000 × *g* for 70 min at 4 °C using an Optima XL-100 K (Beckman Coulter, Inc., Brea, CA, USA) with a swing rotor SW41Ti (Beckman Coulter, Inc.). The residual fraction was washed with phosphate-buffered saline (PBS; pH 7.4) and collected by ultracentrifugation (UC) at 210,000 × *g*. PBS was added to the final sEV-enriched fraction. Proteins were quantified using a Qubit4 system (Thermo Fisher Scientific).

### Collection of sEVs (tangential flow filtration [TTF])

MSCs at 80% confluence were cultured in serum-free DMEM (Thermo Fisher Scientific) with GlutaMAXTM (100 ×) (Thermo Fisher Scientific) for 48 h. The supernatant was collected, centrifuged at 2000 × *g*, and filtered through a 0.22-μm filter (StericupTM Quick Release DuraporeTM, Merck Millipore, Burlington, MA, USA). The filtered supernatant was concentrated using ÄKTA flux™ s (Cytiva, Tokyo, Japan) with a 500-kDa cutoff filter (UFP-500-C-2U; Cytiva) and then diluted with an appropriate volume of PBS.

### Nanoparticle tracking

The number and size of the purified EV particles were measured using a Viewsizer 3000 (HORIBA Scientific, Japan). The particles were irradiated with lasers at three wavelengths, and the Brownian motion of each particle was traced from the scattered light. The particle size and number were measured from the diffusion velocity based on the Stokes–Einstein formula. The fluorescent particles were measured according to the manufacturer’s instructions.

### Culture of mouse macrophages

Bone marrow cells collected from mouse femurs were cultured at 37 °C in the presence of 5% CO_2_ in ultra-low attachment flasks (Corning, NY, USA) and medium (DMEM/F12; Thermo Fisher Scientific) containing 20 ng/ml colony stimulating factor-1 (CSF-1) (Peprotech Inc., Rocky Hill, NJ, USA). The medium was changed twice weekly, as described previously [4]. After 5 or 7 days, the macrophages were harvested.

### Culture of mouse hepatocytes, hepatic stellate cells (HSCs), and liver sinusoidal endothelial cells (LSECs)

HSCs were isolated from the livers of 30-month-old female mice. Hepatocytes and LSECs were isolated from 8- to 16-week-old male mice. Parenchymal and non-parenchymal cell fractions were isolated using liver perfusion medium (Thermo Fisher Scientific) and liver digestive medium (Thermo Fisher Scientific). Hepatocytes were obtained from the parenchymal cell fraction, and HSCs and LSECs were obtained from the non-parenchymal cell fraction [3].

### Cellular uptake of tdTomato-expressing sEVs within liver components: intracellular observations

Each liver cell line was seeded onto chamber slides (Corning). tdTomato expressing sEVs were harvested by UC, and 2 μg/ml of them were added after 12–24 h of cell adhesion. Four hours later, the cells were fixed in 4% paraformaldehyde, stained with 4′,6-diamidino-2-phenylindole, dihydrochloride for nuclear staining, and observed under a BZ-9000 fluorescence microscope (Keyence, Osaka, Japan).

### Uptake of tdTomato-expressing sEVs in the mouse liver cirrhosis model: tissue-level observations

After anesthesia, the inferior vena cava was ligated into the chest, an intravenous line was inserted into the inferior vena cava in the abdomen, and the liver was ligated, leaving part of the right lobe. Thus, a retrograde route was created that could only be used to administer to a portion of the right lobe of the liver (Fig. 3A). The route was used to administer tdTomato expressing sEV alone (20 μg/mouse) or tdTomato expressing sEV (20 μg/mouse) and AlexaFluor 488 (a green fluorescent dye) conjugated F4/80 antibody (final concentration; 25 μg/nl, Abcam, Cambridge, UK). After administration, livers were removed and incubated at 37 °C for 4 h and observed under confocal microscope systems A1R-HD25 and AX R using Vector® TrueVIEW® Autofluorescence Quenching Kit with 4′,6-diamidino-2-phenylindole, dihydrochloride (Vector Laboratories, Burlingame, CA, USA).

### sEV injection into cirrhotic mice

Male mice were intraperitoneally injected twice a week with 1.0 ml/kg carbon tetrachloride (CCl4; Wako Pure Chemical Industries Ltd., Osaka, Japan) dissolved in corn oil (Wako Pure Chemical Industries Ltd., 1:10 v/v) to induce cirrhosis. Eight weeks after the CCl4 injection, PBS (vehicle) and sEVs collected by UC (5, 10, or 15 μg/mouse) or TFF (5 μg/mouse) were injected into the tail vein. Serum and liver fibrosis analyses were performed 4 weeks after injection.

### Western blot

Lysates of sEVs were separated by SDS-PAGE using 4–20% Novex™ 4–20% Tris–Glycine Mini Gels (Thermo Fisher Scientific) and transferred to Trans-Blot® Turbo™ Mini PVDF Transfer Packs (Bio-Rad). Electrophoresis, blotting, and antibody treatment were performed using a Mini Gel Tank (Thermo Fisher Scientific), Pierce Power Blotter Stainer System (Thermo Fisher Scientific), and iBind Western Systems, respectively, following the manufacturer’s instructions. Primary antibodies for western blotting (Exosome-anti CD9, 10626D, dilution:1:50; CD63, 10628D, dilution:1:50; CD81, 10630D, dilution:1:50) were purchased from Thermo Fisher Scientific. Secondary antibodies (anti-mouse IgG horseradish peroxidase-conjugated whole antibodies) were purchased from GE Healthcare (Chicago, IL, USA). All blots were obtained from the same experiment and processed in parallel.

### Serum analyses

Blood samples were obtained from the abdominal aorta of mice 4 weeks after the injection of sEV. Serum alanine aminotransferase (ALT), alkaline phosphatase (ALP), and albumin (ALB) levels were determined by Oriental Yeast Co., Ltd. (Tokyo, Japan).

### Sirius red staining

To quantify fibrosis, liver tissues were fixed with 10% formalin 4 weeks after the injection of sEV. The fixed tissue was cut into 4-μm-thick sections and stained with Sirius Red. Ten images were randomly captured from each section and analyzed using ImageJ software version 1.6.0 20 (National Institutes of Health, Bethesda, MD, USA).

### Hydroxyproline assay

Four weeks after sEV administration, the concentration of the collagen component, hydroxyproline, was measured in the cirrhotic livers. Liver samples (20 mg) were homogenized and subjected to the QuickZyme hydroxyproline assay (QuickZyme Bioscience, Zernikedreef, Netherlands) according to the manufacturer’s protocol.

### Statistical analyses

Data were processed using GraphPad Prism v. 9.3.1 (GraphPad Software Inc., La Jolla, CA, USA) and are presented as means ± standard errors of the means. Data were assessed using the Mann-Whitney *U* test. Differences were considered statistically significant at *p* < 0.05.

## Results

### Successful creation of extracellular vesicles with td-Tomato to confirm the dynamics of extracellular vesicles

**Fig. 1:**
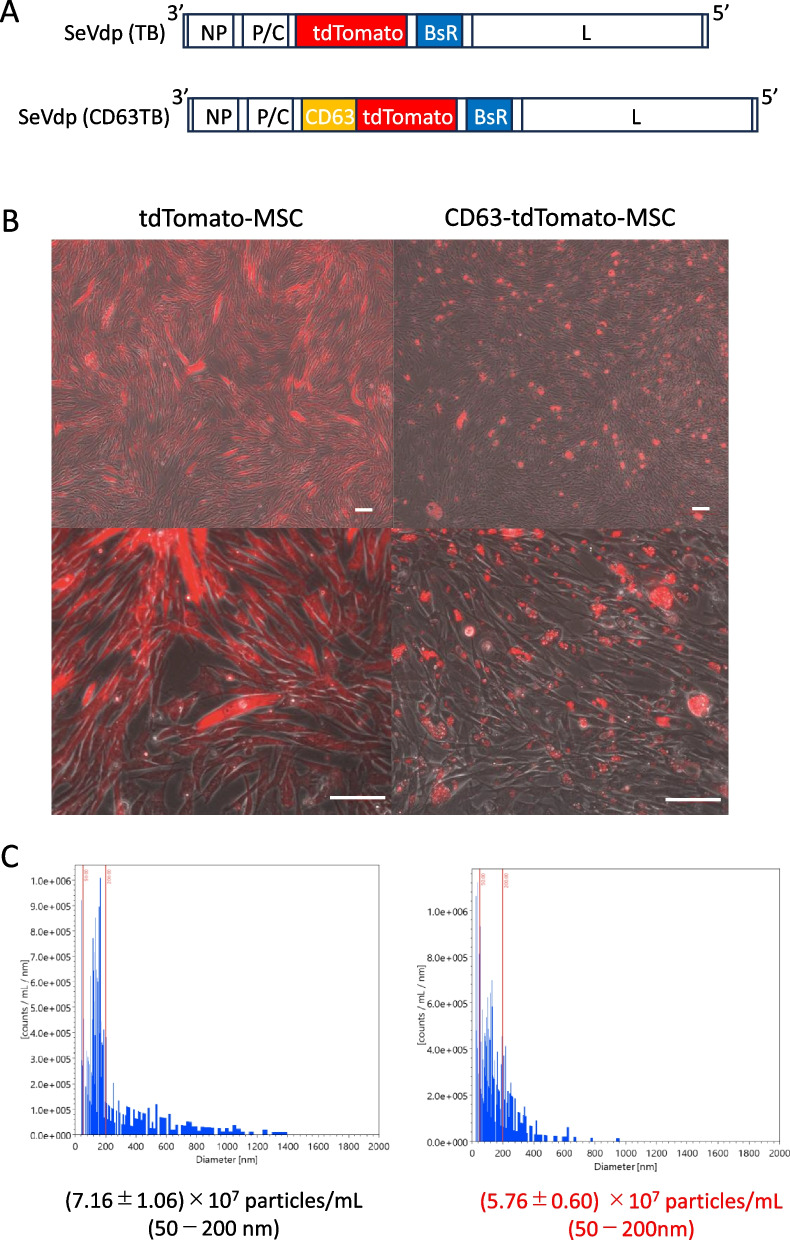
Creating tdTomato expressing small extracellular vesicles (sEVs). **A** Vector structure of SeVdp (TB) or SeVdp (CD63TB) expressing a fusion protein of tdTomato only or CD63 and tdTomato, respectively. **B** Cultured mesenchymal stem cells (MSCs) infected with SeVdp (TB) (left upper and lower panels) or SeVdp (CD63TB) (right upper and lower panels). Scale bar, 100 μm. **C** The representative total number of particles (left panel) and tdTomato-expressing particles (right panel) from MSCs infected with SeVdp (CD63TB). *n* = 3 per experiment

To date, no study has confirmed in detail which cells tend to take up sEVs among liver constituent cells or in an actual liver cirrhosis model. This is because sEVs are small and difficult to visualize. In addition, many studies have been conducted using a method to recognize extracellular vesicles using exogenous labeling dyes. However, we encountered many false positives in our study, and the need to further improve the accuracy of the experiment was considered. In contrast, studies have been conducted using the IVIS Imaging System to deposit luciferase on extracellular vesicles and observe their dynamics, and a high accumulation of luciferase in the liver has been reported. Nevertheless, by means of IVIS-based methods, it remains unclear which cells in the liver take up most sEVs or through where the sEVs enter the damaged cirrhotic liver. The MSCs employed in this study were isolated from human adipose tissue, and CD63 serves as a common marker for sEVs. Consequently, the successful engineering of sEVs was accomplished by integrating tdTomato, a red fluorescent dye, into CD63 through the utilization of Sendai virus vectors (CD63-tdTomato-MSC) (Fig. 1A, B). The transduction efficiency prior to selection by blasticidin S was 91.64 ± 1.22%. Following selection with blasticidin S, a transduced cell population was observed across all cells. From an approximate 3 × 10^7^ cell population, the resulting EV count from CD63-tdTomato-MSC culture supernatant was (7.16 ± 1.06) × 10^7^ particles/mL of total EVs and (5.76 ± 0.60) × 10^7^ particles/mL for red fluorescent positive EVs (comprising 88.6 ± 3.8% of the red fluorescent positive fraction) within 75 ml of the supernatant (Fig. 1C).

### sEVs of MSCs are highly engulfed by macrophages in vitro, in the cirrhosis model, and in the damaged area where fibrosis occurs

**Fig. 2:**
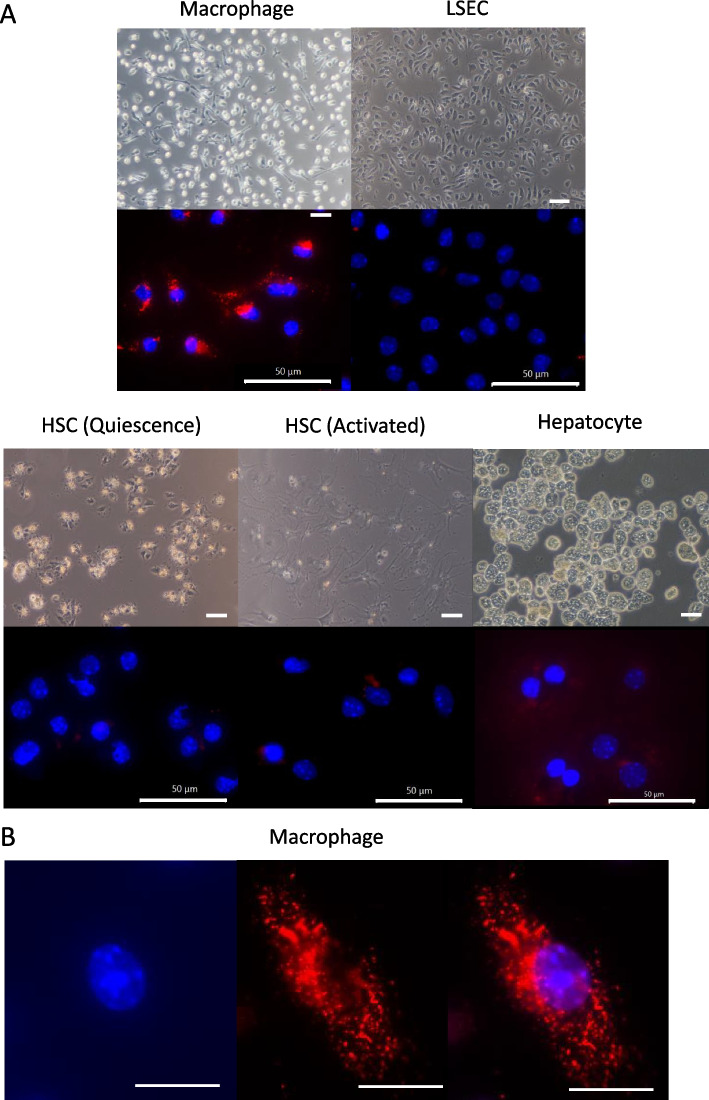
Uptake of tdTomato expressing small extracellular vesicles (sEVs) into liver constituent cells. **A** Uptake by macrophages, liver sinusoidal endothelial cells (LSEC), quiescent hepatic stellate cells (HSCs), activated HSCs, and hepatocytes in vitro. Upper panels: normal observation images, lower panels: fluorescence images. 2.0 μg/mL of tdTomato expressing sEVs were added 4 h before the observation. Nuclei were stained with DAPI. Scale bar, 50 μm. *n* = 3 per experiment. **B** Magnified view of the macrophages expressing tdTomato sEVs. Scale bar, 20 μm. Left panel: DAPI, middle panel: tdTomato, right panel: merged view

**Fig. 3:**
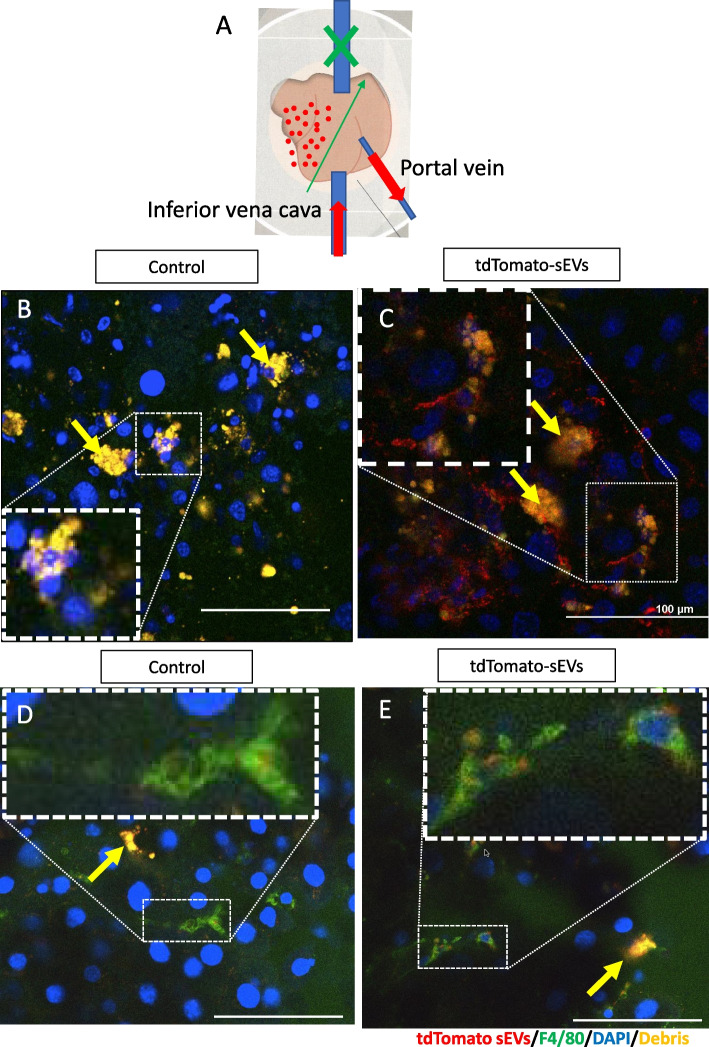
Uptake of tdTomato expressing small extracellular vesicles (sEVs) into cirrhotic livers. **A** Schematic of injection of tdTomato expressing sEVs in the part of the right lobe of murine cirrhotic livers. Red arrows show the flow of injected sEVs. Green X and arrows show ligatures. Red dots shows the area of sEVs injection. **B**, **C** Observations after administration of control sEVs (**B**) and tdTomato expressing sEVs (**C**) in the 8 weeks CCl4 damaged livers. **D**, **E** Observations after administration of control sEVs (**D**) and tdTomato-expressing sEVs (**E**) when macrophages were made recognizable by AlexaFluor 488 (green) in the 8 weeks CCl4 damaged livers. 400 μl of control sEVs or tdTomato-expressing sEVs (concentration; 20 μg/mL) were injected from inferior vena cava 4 h before observation. Yellow arrows show the hepatocyte debris, which is detected in the damaged area of the liver. Scale bar, 100 μm. *n* = 3 per experiment

We verified the uptake of tdTomato-expressing sEVs by primary cultured macrophages, LSECs, HSCs (quiescent and activated forms), and hepatocytes (Fig. 2A, B). Our results showed that sEVs were taken up in overwhelming amounts by macrophages. Next, we verified the location and cell type of uptake in the livers of cirrhotic mice induced with CCl_4_. Visualization was difficult with tail vein administration alone. Therefore, we ligated the hepatic vessels in the liver and devised a method to administer sEVs only to small right lobes so that high concentrations of sEVs could accumulate in these lobes (Fig. 3A). Our results indicated robust sEVs accumulation strongly in regions displaying damage and hepatocellular debris (Fig. 3B, C). We then administered AlexaFluor 488 conjugated F4/80 antibody and tdTomato-expressing sEVs simultaneously so that the macrophages could be recognized by the green fluorescent dye and analyzed. The results showed that a large number of sEVs were taken up by macrophages in areas damaged by fibrosis and hepatocellular debris (Fig. 3D, E).

### sEVs do not enhance treatment in a volume-dependent manner

**Fig. 4:**
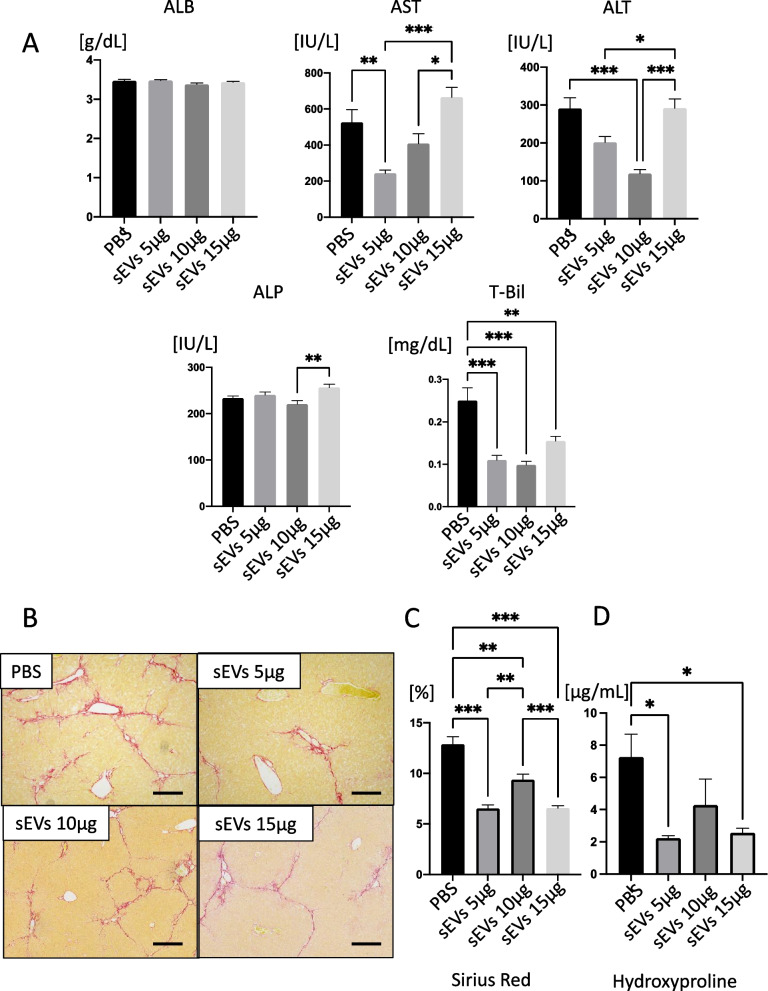
Verification of the difference in the therapeutic effect of different doses of small extracellular vesicles (sEVs). Eight weeks after the CCl4 injection, PBS (vehicle) and sEVs collected by ultracentrifugation (5, 10, or 15 μg/mouse) were injected into the tail vein. Serum and liver fibrosis analyses were performed 4 weeks after injection. **A** Serum levels of albumin (ALB), aspartate transaminase (AST), alanine transaminase (ALT), alkaline phosphatase (ALP), and total bilirubin (T-Bil). **B** Sirius Red staining of liver tissues. Scale bar, 500 μm. **C** Calculation of Sirius Red staining area and **D** quantification of hydroxyproline levels. Data are presented as the mean ± SEM; *n* = 6 per experiment. *p < 0.05, **p < 0.01, and ***p < 0.001, Mann‒Whitney *U* analysis

Previously, we administered 5 μg of sEVs/mouse in the mouse model of CCl_4_ liver cirrhosis and observed its therapeutic effects. Next, we compared the therapeutic efficacy of 5 μg, 10 μg, and 15 μg of the ultracentrifuged sEV doses with that of the PBS-only control group and examined whether there were any visible differences in safety. The mice used in this study were the same CCl_4_ cirrhosis model mice described previously. Our results demonstrated that all doses were administered safely without manifesting any pulmonary embolic symptoms or other signs of cellular overdose. No other discernible abnormalities were observed. Regarding therapeutic effect, T-Bil showed a decreasing trend in all groups, whereas AST and ALT in the 5- and 10-μg groups were lower than those in the control group; however, there was little improvement in the 15-μg group (Fig. 4A). Thus, there was no evidence of dose dependency in these 5–15 μg groups. We also performed Sirius Red staining (Fig. 4B, C) and quantification of hydroxyproline (Fig. 4D) to determine whether there was any improvement in fibrosis. The results showed that fibrosis was improved in all three groups compared with the controls; however, fibrosis did not improve in a dose-dependent manner in the sEV groups, indicating that the therapeutic effects of sEVs are not necessarily dose-dependent.

### sEVs can be harvested by TTF with comparable therapeutic efficacy

**Fig. 5:**
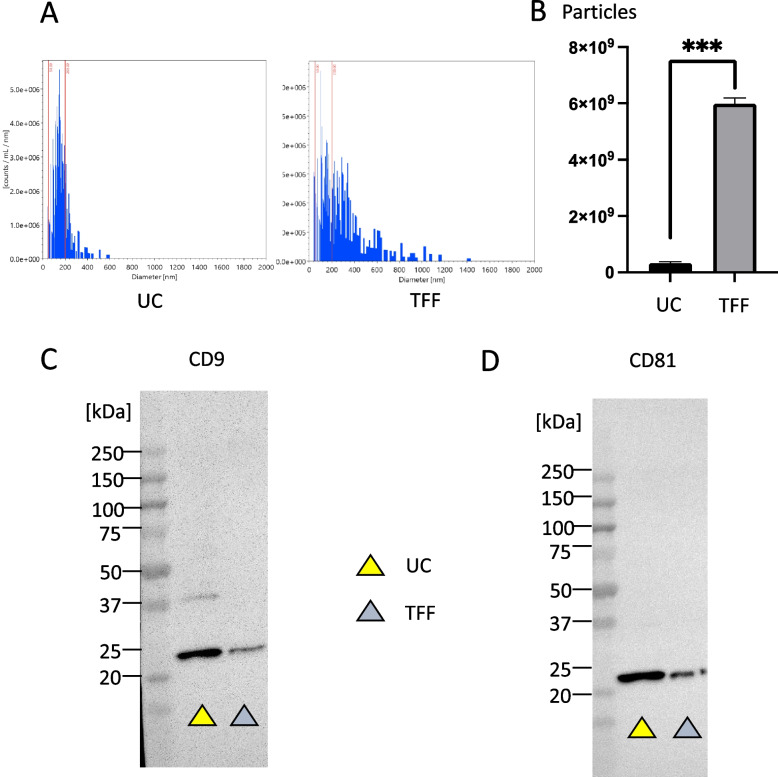
Collection of small extracellular vesicles (sEVs) by ultracentrifugation (UC) and tangential flow filtration (TTF). **A** Particle size distribution of sEVs when collected by UC and TFF. **B** Particle number collected from the same volume (200 ml) of mesenchymal stem cell (MSC) culture supernatant. Western blot analysis of CD9 (**C**), and CD81 (**D**) of sEVs collected using UC and TFF. *n* = 3 per experiment. ***p < 0.001, Mann–Whitney* U* analysis

**Fig. 6:**
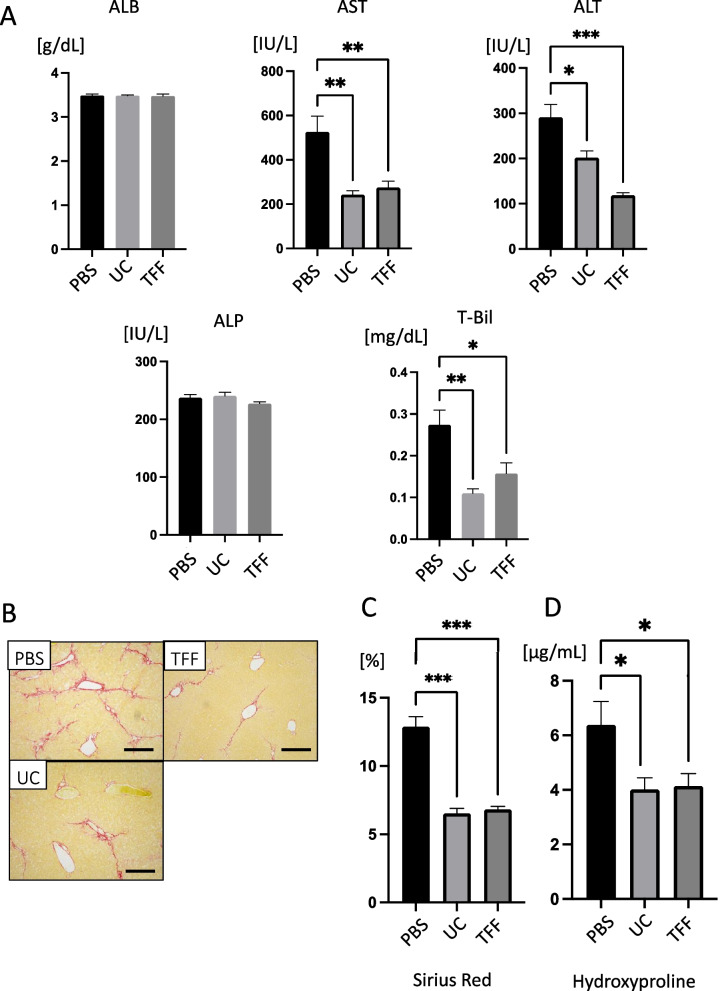
Therapeutic effect of small extracellular vesicles (sEVs) when collected by ultracentrifugation (UC) and tangential flow filtration (TFF). Eight weeks after the CCl4 injection, PBS (vehicle) and sEVs collected by UC (5 μg/mouse) or TFF (5 μg/mouse) were injected into the tail vein. Serum and liver fibrosis analyses were performed 4 weeks after injection. **A** Serum levels of albumin (ALB), aspartate transaminase (AST), alanine transaminase (ALT), alkaline phosphatase (ALP), and total bilirubin (T-Bil). **B** Sirius Red staining of liver tissues. Scale bar, 500 μm. **C** Calculation of Sirius Red staining area and **D** quantification of hydroxyproline levels. Data are presented as the mean ± SEM; *n* = 6 per experiment. *p < 0.05, **p < 0.01, and ***p < 0.001, Mann–Whitney *U* analysis

We investigated whether sEVs could be extracted from culture supernatants using laboratory-scale equipment capable of performing TFF. While the size of the vesicles collected using TFF (TFF group) was marginally larger than those collected using ultracentrifugation (UC group), TFF exhibited the advantage of reduced procedural time alongside enhanced sEV yield. Specifically, when collecting with 200 ml of supernatant, TFF yielded a 0.40 ± 0.01 reduction in procedural time and a 19.45 ± 2.95-fold increase in the final particle count (Fig. 5A, B) (*p* < 0.001). The sEVs collected were confirmed to contain CD9 and CD81 in both the TF and UC groups using western blotting (Fig. 5C, D). Next, we decided to see the effect of the collection method on treatment efficacy; 5 μg of sEVs collected from the TFF and UC groups were administered to the cirrhosis model mice (8-week-old wild-type C57BL/6 mice were used. The mice were injected with CCl_4_ twice weekly for 12 weeks. A single dose of sEVs was administered at 8 weeks, and the mice were analyzed at 12 weeks after the CCl_4_ injection). The results were compared with those of the control group treated with PBS, which revealed that AST, ALT, and T-Bil levels in both the TFF and UC groups tended to improve than those in the control group, with no significant differences between the TTF and UC groups (Fig. 6A). Sirius Red analysis (Fig. 6B, C) and hydroxyproline quantification (Fig. 6D) were performed to determine whether there was any improvement in fibrosis. Both the TFF and UC groups showed significant improvement compared with the control group, and there were no significant differences between the TTF and UC groups. This indicates that collection via TFF does not affect the therapeutic efficacy of sEVs, and that collection methods other than UC can dramatically improve the time and capacity of the collection process.

## Discussion

In this study, we analyzed three points that were unclear regarding the development of liver cirrhosis treatment using EVs from MSCs: the distribution of sEVs, verification of their efficacy in methods of mass collection, and their volume dependency. We found that sEVs of MSCs accumulated mostly in macrophages in the damaged areas of the liver. In addition, although the UC method was commonly used to collect sEVs for research purposes, we verified that TFF could be used for efficient collection and that the effectiveness of TFF is not reduced. Furthermore, the therapeutic effect of sEVs was not necessarily dose-dependent, and it reached a plateau when the dosage exceeded a certain level.

Warnecke et al. reported the first-in-human clinical trial of the local treatment of cochlear implantation with EVs of MSCs derived from umbilical cord tissue [8]. Treatment with EVs derived from MSCs has been investigated in many areas, including diabetes, kidney, eye, stroke, and graft-versus-host disease [9, 10]. Although there is already considerable clinical experience regarding the administration of MSCs, there is a risk of embolization if large amounts are administered, such as in cases when they are trapped in the lungs. However, EVs, which are very small particles, are less likely to cause embolization and are more easily transferred to the target organs. However, EV therapy has the following limitations.

One primary limitation pertains to mass culture and the preservation of cell identity within such cultures. As primary MSCs inherently possess finite lifespans and tend to deteriorate or senesce over time, their functionality can also exhibit variability based on donor factors and culture conditions. Therefore, one potential approach to overcome this limitation is the exploration of immortalization techniques.

Significant work remains to be undertaken to determine the kind of EVs that would be most suitable for the treatment of cirrhosis. We hypothesized that macrophages, which have anti-inflammatory properties, phagocytosis, motility, and produce matrix metalloproteinase, may accumulate in liver lesions and exert housekeeping and fibrinolytic effects, leading to a therapeutic effect [4, 6, 11, 12]. Therefore, we focused on elements such as annexin-A1, aminopeptidase-N, and lactotransferrin based on proteomic analysis of extracellular vesicles obtained after stimulation of MSCs with IFN-γ, which showed particularly good therapeutic effects [6]. However, since miRNAs and other nucleic acids may influence treatment, as well as the possibility that many substances may be involved in the function, the search for specific effective substances will continue in the future. Nevertheless, sEVs stand as promising candidates for effective transport of active substances within medical contexts.

In addition to mass culture, extraction methods are also important. Busatto et al. reported the usefulness of TFF [13] and Visan et al. reported that TFF and size-exclusion chromatography can be used for efficient recovery [14]. In addition, Warnecke et al. used TFF as the collection method in their first-in-human clinical trial [8]. We found that the therapeutic effect of sEVs obtained via TFF was comparable with that of those obtained via the UC method. Nevertheless, a marginal disparity in particle sizes emerged between the methodologies. Unfortunately, we were unable to ascertain whether variations in EV size correspond to changes in contents and treatment outcomes, an inherent limitation of this study. In addition, our investigation unveiled that the treatment effect did not exhibit dose dependency. We speculate that this phenomenon may be attributed to an effect saturation point when the concentration of sEV-affected cells surpasses a certain threshold. However, conclusive validation remains pending.

Some studies have summarized the distribution of EVs, indicating that the liver, along with the lungs, exhibits a propensity for accumulating these vesicles within a span of 12 to 24 h. Scavenger receptors are important for the uptake of EVs, and phosphatidylserine, which is expressed in several EVs, has also been reported to be important for uptake [15, 16]. In this study, we generated EVs expressing tdTomato and showed that tdTomato accumulated in liver lesions and that macrophages had the highest accumulation of tdTomato in vitro and in vivo. To our knowledge, this is the first report on the localization of sEVs of MSCs in the liver and their uptake by cells. These results support our hypothesis that sEVs of MSCs exert therapeutic effects on cirrhosis via macrophages. Therefore, targeting macrophages in the liver may be an effective treatment for cirrhosis using sEVs.

Ensuring the preservation of EVs holds substantial importance in clinical practice. Furthermore, various potential strategies exist to enhance the therapeutic effect of EVs. These encompass the utilization of natural-type EVs, collected directly from cell supernatants without any modification or pre-conditioning, such as MSCs stimulated with IFN-γ, as we have previously employed [6]. Moreover, other approaches to enhance therapeutic effect involve modifying cells, such as introducing miRNAs or proteins into cells through gene transfer or altering the cell surface properties to resemble those of the target cells. In this study, we introduced tdTomato into extracellular vesicles by fusing it with CD63. The incorporation of tdTomato serves the dual purpose of visualizing sEVs and opens up the potential for introducing specific proteins within sEVs. These are essential considerations that we have not yet thoroughly explored at this juncture.

Additionally, there is still the issue of regulation as a limitation of the application of EVs. While regulatory issues will be further discussed and matured in the future, some opinions are presented below.

Lim et al. reported that functional heterogeneity among primary MSC extends to the functionality of secreted EVs and compromises lot-to-lot consistency in the quality and performance of the EVs [17]. The Japanese Society for Regenerative Medicine has pointed out the parallels between EVs and processed cells in terms of manufacturing processes and safety concerns, emphasizing that as EVs are released from cells, similar quality control strategies should be devised to ensure the quality of the final product (EVs) by regulating the raw materials and manufacturing procedures [18].

Although there are still issues to be resolved, we believe that once the issues of cell quality and mass culture are resolved, this field will move toward clinical application and may apply to many diseases, in addition to liver diseases.

## Conclusion

In this study, we identified some unknown aspects regarding the dynamics, collection, and capacity dependence of sEVs. Our findings shed light on the crucial role of macrophages as a significant target for sEVs. However, it is noteworthy that the therapeutic effect of sEVs does not exhibit a linear increase with higher volumes. Furthermore, our study points towards the future possibility of enhancing the efficiency of sEVs collection. Overall, our research contributes to a better understanding of these intricate dynamics and holds potential implications for their therapeutic applications.

### Supplementary Information

**Additional file 1.**

## Data Availability

All data required to evaluate the conclusions of this study are provided in the main text of the manuscript. This study included no data deposited in external repositories. Additional data related to this study may be requested from the authors.

## Abbreviations

AD-MSC: Adipose tissue-derived mesenchymal stem cell
ALB: Albumin
ALP: Alkaline phosphatase
ALT: Alanine aminotransferase
AST: Aspartate aminotransferase
CCl_4_: Carbon tetrachloride
DMEM: Dulbecco’s modified Eagle’s medium
EVs: Extracellular vesicles
FBS: Fetal bovine serum
HSCs: Hepatic stellate cells
IFN-γ: Interferon-gamma
LSEC: Liver sinusoidal endothelial cell
miRNA: Micro ribonucleic acid
MSC: Mesenchymal stem cell
PBS: Phosphate-buffered saline
PI: Propidium iodide
sEV: Small extracellular vesicle
T-Bil: Total bilirubin
TFF: Tangential flow filtration
UC: Ultracentrifugation
