# Supplementary figures and images for: Analysis of distribution, collection, and confirmation of capacity dependency of small extracellular vesicles toward a therapy for liver cirrhosis
Source: Inflamm Regen. 2023 Oct 9;43:48. doi: 10.1186/s41232-023-00299-x (PMC10561446; doi:10.1186/s41232-023-00299-x)

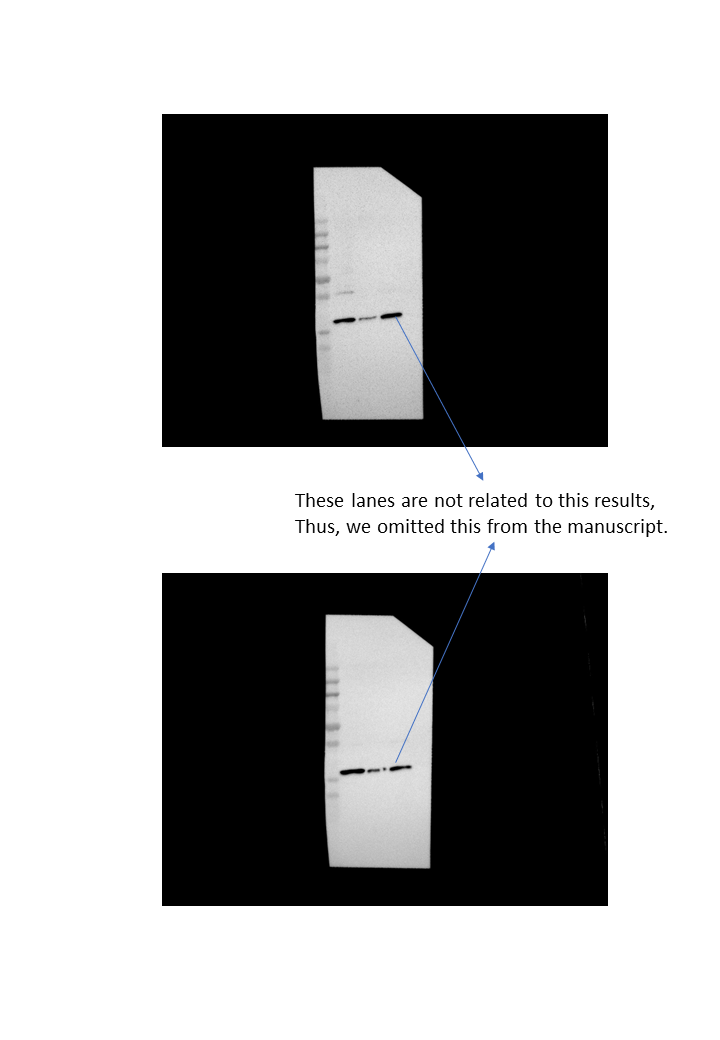

Supplement: Supplementary file 1 — Additional file 1. [file 41232_2023_299_MOESM1_ESM.tif]
